# Listening to Unheard Voices: Addressing Systemic Racism to Improve Maternity Care for Black Women After Perinatal Loss

**DOI:** 10.3390/ijerph23050572

**Published:** 2026-04-28

**Authors:** Jeri M. Antilla, Linda M. DiClemente, Amy C. Buckenmeyer, Aubree Villarreal, Nicole Rek

**Affiliations:** 1 School of Nursing, University of Michigan, Ann Arbor, MI 48109, USA; lmdiclem@umich.edu (L.M.D.); buckena@umich.edu (A.C.B.); nrek@umich.edu (N.R.); 2 College of Literature, Science, and the Arts, University of Michigan, Ann Arbor, MI 48109, USA; aubreev@umich.edu

**Keywords:** perinatal loss, systemic racism, maternal health, patient–provider dynamics, maternity care quality

## Abstract

**Highlights:**

**Public health relevance—How does this work relate to a public health issue?**

- This study explores how systemic racism and patient–provider dynamics shape Black women’s maternity care during and after perinatal loss.
- It links perinatal loss to public health concerns, including psychological distress, mistrust, and barriers to follow-up maternity care.

**Public health significance—Why is this work of significance to public health?**

- This study shows that dismissive communication, racialized assumptions, and gaps in follow-up care intensify distress after perinatal loss.
- It shows that these experiences can shift the burden of self-advocacy onto Black women and shape care-seeking in later pregnancies.

**Public health implications—What are the key implications or messages for practitioners, policy makers and/or researchers in public health?**

- Public health and maternity care systems should strengthen follow-up after perinatal loss and improve coordination across maternity, mental health, and bereavement care.
- Healthcare providers, policy makers, and researchers should support culturally safe care and evaluate continuity-of-care models, navigation support, doula access, and ongoing training and assessment to address racial bias and strengthen cultural safety.

**Abstract:**

Black women in the United States experience inequities in perinatal and neonatal mortality, contributing to psychological stress during and after perinatal loss. This analysis drew on a subset of interviews from a larger qualitative dataset and explored the experiences of 22 Black women who experienced perinatal loss and were pregnant or had given birth after a loss, focusing on feeling unheard by healthcare providers. Semi-structured interviews were conducted, and data were analyzed using descriptive coding and inductive thematic analysis. Three themes emerged: unheard and dismissed concerns, biased and stratified care, and perinatal loss follow-up gaps driving self-advocacy. Women described how systemic racism intensified psychological distress, expressed as heightened anxiety and uncertainty in subsequent pregnancies after perinatal loss. Findings underscore the need for maternity settings to confront racial bias and strengthen cultural safety. Care environments that validate Black women’s concerns and act on them may help rebuild trust and improve maternal and newborn outcomes. The study calls for changes in maternity and mental healthcare aimed at addressing systemic racism and strengthening culturally responsive, equitable care. These findings have implications for perinatal public health practice and policy, including surveillance, prevention, and community-responsive approaches to maternity care during and after perinatal loss.

## 1. Introduction

In the United States (U.S.), Black women continue to experience profound inequities in maternal health, including a substantially greater risk of pregnancy-related death than White women, with recent estimates showing 50.3 deaths per 100,000 live births among Black women compared with 14.5 deaths among White women [1]. Black women also experience inequities across pregnancy, birth, and postpartum care, including differential treatment, delayed evaluation of symptoms, and reduced access to timely, high-quality maternity care [2]. These disparities arise from multiple intersecting factors including racism, chronic stressors across the life course, maternal comorbidities, health behaviors, socioeconomic conditions, and inequities in access to quality maternity care [3,4]. Prior work has consistently documented perceived racial discrimination across the pregnancy–postpartum continuum [5,6,7]. Black women have described having concerns dismissed or left unaddressed and receiving racially biased care from healthcare providers, all of which may compromise health and pregnancy outcomes [5,6,8]. Additionally, they are less likely to receive timely and adequate prenatal care [9,10], which increases the risk of adverse pregnancy outcomes such as preterm birth, low birth weight, infant mortality, and perinatal loss [11]. These inequities also raise concerns about the quality and experience of maternity care that Black women receive.

According to the Association of Women’s Health, Obstetric and Neonatal Nurses (AWHONN) [12], respectful maternity care is person-centered care that upholds dignity, equity, and empathy. The Centers for Disease Control and Prevention (CDC) [13] further defines respectful maternity care as care that is free from harm and mistreatment, supports shared decision-making, and helps women feel heard and respected. The effects of inadequate maternity care may extend beyond physical outcomes to influence how women understand, remember, and respond to maternity care during and after perinatal loss. Experiences of dismissal, discrimination, and poor-quality care may intensify the emotional and psychological effects of perinatal loss, and shape whether women feel safe seeking or trusting care in a subsequent pregnancy [13,14].

Perinatal loss is often used as an umbrella term for loss during pregnancy and the newborn period, including miscarriage, stillbirth, and neonatal death [15,16]. In this article, we focus on later pregnancy and neonatal losses.

These inequities also extend across adverse perinatal outcomes. Preterm birth, defined as birth before 37 weeks’ gestation, affects roughly 1 in 10 infants [17], with Black women experiencing a higher preterm birth rate (14.6%) than White women (9.4%) [18]. Disparities also extend to fetal and infant mortality and to low birthweight (LBW, <2500 g) and very low birthweight (VLBW, <1500 g). In 2021, fetal mortality was 9.9 per 1000 live births among Black women compared with 4.8 among White women [19,20]. Infant mortality among Black infants also remained more than twice the rate observed among White infants [19,20].

Social drivers of health (SDoH) and chronic stress exposure contribute to these inequities [21,22,23,24]. Ongoing stress can intensify when Black women’s concerns are minimized, met with nonresponsive communication, or filtered through racialized assumptions in clinical assessment. Barriers to access and continuity of care may further compound the burden of navigating maternity care. After perinatal loss, limited proactive follow-up care from healthcare providers may further heighten Black women’s distress and require their sustained self-advocacy to obtain answers and responsive, culturally safe maternity care. These cumulative experiences can increase allostatic load, disrupt physiologic regulation, and contribute to adverse pregnancy outcomes [25,26,27]. Allostasis describes the body’s adaptation to stress through physiological adjustment, and it helps explain how repeated stressors can strain homeostatic systems over time [27]. This biologic framework aligns with the “weathering” hypothesis, which situates stress within social conditions, and proposes that cumulative disadvantage accelerates health deterioration, increasing risk for hypertension, LBW, VLBW, and preterm birth [28]. Persistent stress can elevate cortisol and norepinephrine levels, which may contribute to preterm labor and compromised fetal development [29,30].

Despite growing evidence linking systemic racism, healthcare provider bias, and SDoH to adverse pregnancy outcomes, important gaps remain in understanding how Black women experience these dynamics before and after perinatal loss and in subsequent pregnancies [31,32,33,34,35,36]. Limited research has examined how dismissive or discriminatory care and gaps in follow-up care during and after perinatal loss shape psychological distress and later engagement with maternity care, especially among Black women [34,37,38,39]. This study aimed to explore the experiences of Black women in the U.S. who identified systemic racism and patient–provider dynamics as factors that shaped their maternity care before and after perinatal loss, and during a subsequent pregnancy. This analysis draws on a subset of interviews from a larger qualitative dataset focused on chronic psychological stressors, with additional thematic findings published separately [3]. We do not suggest these patterns occur exclusively among Black women; rather, we interpret the findings within U.S. maternity care contexts that disproportionately shape Black women’s experiences, including racialized clinical interactions and structural conditions that influence access, continuity of care, and support.

## 2. Materials and Methods

A total of 22 Black women residing in the U.S. were included in this qualitative study. Each woman had previously experienced perinatal loss and was either pregnant at the time of the interview or had given birth after the loss. Approximately 80% of miscarriages occur in the first trimester [40]; for this reason, the study operationalized the qualifying loss as one occurring from the second trimester (14 weeks’ gestation) through neonatal death within the first 28 days of life. This approach narrowed the analytic sample to later pregnancy and neonatal losses and aligned with the study’s eligibility criteria.

To determine eligibility for the study, the inclusion criteria were as follows: women who self-identified as Black, were at least 18 years of age, and could read and speak English. Women were required to have experienced at least one qualifying fetal or neonatal loss between 14 weeks’ gestation and 28 days of life. In addition, they were either pregnant at the time of participation or had given birth after their loss. Finally, consistent with the eligibility criteria of the larger qualitative dataset from which this analysis was drawn, women reported exposure to stress-related circumstances during the perinatal loss period and/or in the time leading up to and during a subsequent pregnancy.

### 2.1. Setting and Design

This study used Black feminist thought and a life-course perspective to examine how systemic racism and patient–provider dynamics shape Black women’s experiences of perinatal loss and subsequent pregnancy. Black feminist thought foregrounds Black women’s perspectives and emphasizes how intersecting structures of power influence healthcare interactions and inequities, including racialized assumptions and differential treatment in clinical settings [41]. Consistent with this framework, race-neutral approaches may fail to capture the layered ways Black women experience systemic racism and inequitable maternity care [42]. A life-course perspective supports examination of how the conditions in which people are born, raised, live, work, and age shape health trajectories and perinatal outcomes across the lifespan [29,43,44]. Lu and Halfon [29] argue that inequities in pregnancy and birth outcomes reflect early-life exposures and the cumulative burden of stress over time. Together, these frameworks guided our interpretation of how being unheard, navigating biased and stratified care, and experiencing limited follow-up care during and after perinatal loss may contribute to psychological distress and influence maternity care experiences after perinatal loss and during a subsequent pregnancy [42]. Taken together, these frameworks offer an integrated lens for examining the layered, lived realities Black women navigate in maternity care and perinatal outcomes. For women in this study, that lens also helps explain why culturally concordant or culturally safe care may matter, as broader histories of systemic racism and inequitable treatment in maternity care settings shape trust, rapport, and perceived understanding [42].

This study used an interpretive phenomenological design. We drew on phenomenology to center women’s lived experiences and the meanings they assigned to those experiences [45,46]. This design aligned with the study’s aim to explore the experiences of Black women in the U.S. who identified systemic racism and patient–provider dynamics as factors that shaped their maternity care before and after perinatal loss, and during a subsequent pregnancy.

### 2.2. Sample

Before data collection began, the primary investigator (PI) obtained Institutional Review Board approval through their institution (University of Wisconsin-Milwaukee; IRB ID #19.A.276). Recruitment used snowball sampling and multiple community and clinical outreach channels across the U.S. Flyers were posted or shared through women’s health clinics, perinatal loss support groups, community centers, churches, hair salons, and social media. Women who were interested contacted the PI by email, and the PI then confirmed their eligibility. Eligible women completed electronic informed consent prior to participating. The PI scheduled and conducted the semi-structured, individual interviews. Recruitment continued until additional interviews no longer materially expanded the coding framework or thematic patterning relevant to the analytic focus.

Immediately prior to each interview, the PI reviewed the study process with the women, including what their participation would entail, any foreseeable risks, and the option to withdraw from the study at any time without penalty. The PI also explained the plan to audio-record the interview and how the research team would use the recordings. Each interview began with a brief demographic questionnaire that captured age, geographic region, education, income, employment, relationship status, housing circumstances, and health insurance coverage.

The PI conducted interviews at a scheduled time, either face-to-face or remotely via each woman’s preferred format (e.g., FaceTime, Skype, or telephone). To protect confidentiality while linking demographic data to interview materials, the PI assigned each woman a numeric identifier and maintained all records in de-identified form. Interviews were audio-recorded and lasted approximately 50 min on average. Following completion of the interview, each woman received a $25 gift card in appreciation for her time.

The study included 22 Black women aged 18 to 48 years (M = 34.2, SD = 8.3). Women lived in multiple U.S. regions, with the largest group residing in the Midwest (*n* = 10). The remaining women were located in the Northeast (*n* = 3), Central (*n* = 1), West (*n* = 2), and South (*n* = 6).

Women reported a median annual income of $41,500, with substantial variation across the sample. Seven women reported annual earnings below $20,000, and six reported incomes above $80,000. Employment also varied: sixteen women worked, five were not employed, and one attended school full-time. Educational attainment ranged from less than high school (*n* = 1) to high school completion (*n* = 4), some college (*n* = 8), and a college degree (*n* = 9).

Women described a range of relationship statuses, including married (*n* = 9), single (*n* = 7), divorced (*n* = 4), in a relationship (*n* = 1), and engaged (*n* = 1). Data collection did not capture sexual orientation, partner gender identity, or infertility history, given that the analysis did not examine these domains. Women also reported varied housing circumstances. Nine owned a home, thirteen rented, and six experienced housing instability or homelessness at some point during the pregnancy that ended in loss.

Women reflected variability in insurance coverage, socioeconomic circumstances, and reproductive and loss histories. Twelve women relied on a federal–state insurance program, and ten had private insurance. Women reported between one and eight perinatal losses (M = 2.1, SD = 1.6). Nine women described experiencing one pregnancy loss, four described two pregnancy losses, and four described three pregnancy losses, while one woman reported four pregnancy losses and one reported eight pregnancy losses. Three women also experienced neonatal death within the first 28 days of life. These broader self-reported reproductive and loss histories were not limited to the qualifying loss used for study eligibility. Table 1 therefore includes information from women’s broader reproductive and loss histories, broadening the range of perspectives represented and informing interpretation of transferability, although the current analysis did not permit in-depth comparison across these dimensions.

We collected data through semi-structured, individual interviews. The PI used open-ended prompts and asked follow-up questions in response to what women shared. This flexible format allowed the interviewer to move beyond the guide when needed to clarify meaning and better capture women’s narratives [47,48]. We developed an interview guide based on prior literature to support consistent data collection across interviews. Using this approach, we obtained detailed accounts of Black women’s maternity care experiences surrounding perinatal loss and subsequent pregnancy. Table 2 presents the key topics and prompting questions. The topics summarized in Table 2 address daily stress, perinatal loss, follow-up care, and subsequent pregnancy, supporting analysis of maternity care experiences across interconnected clinical and social contexts.

Field notes were collected throughout the study to supplement the interview data. The PI recorded notes before, during, and after each interview to document situational details, initial impressions, and topics requiring follow-up. These observations supported the interpretation of how women described stress and navigated patient–provider interactions during perinatal loss and in subsequent pregnancies. In qualitative research, field notes enrich interview data by adding detail that strengthens analytic depth [49].

### 2.3. Analysis

Verbatim transcripts were generated from all audio recordings using a professional transcription service. We checked each transcript against the corresponding audio recording, corrected discrepancies, and finalized the text for analysis. To protect confidentiality, we removed identifying information prior to analysis. We then imported transcripts into NVivo 12 (QSR International) for data management and organization, and the PI completed all qualitative coding.

We analyzed the data using inductive thematic analysis, beginning with descriptive coding to label segments of text with short words or phrases that summarized each segment’s primary topic [50,51]. We then compared codes across transcripts, grouped related codes into broader patterns, and iteratively developed themes and subthemes through team discussion [50]. Through this process, we developed three themes: unheard and dismissed concerns, biased and stratified care, and perinatal loss follow-up gaps driving self-advocacy. Throughout the analysis, we met regularly via Zoom to examine code meaning, refine theme boundaries, and ensure that the analysis remained grounded in women’s accounts and consistent with the study’s aim and conceptual framing.

We drew on a larger qualitative dataset focused on chronic psychological stressors, which generated multiple areas of inquiry, including mental wellbeing, coping, and patient–provider dynamics. The present analysis focuses specifically on patient–provider dynamics in maternity care, and findings related to other thematic areas appear in separate publications [3,52]. Some methodological details necessarily resemble the prior publication because both manuscripts rely on the same sampling, recruitment, and interview procedures. This analysis addresses a distinct question related to patient–provider dynamics in maternity care during and after perinatal loss.

#### Rigor

We used multiple strategies to enhance trustworthiness. To support confirmability, the PI maintained a reflexive journal throughout data collection and analysis. This process promoted transparency and helped mitigate researcher bias by documenting the PI’s positionality as a middle-class White woman, a qualitative nurse researcher, and an obstetric nurse with 26 years of clinical experience, as well as her outsider relationship to Black women’s lived experiences of perinatal loss. We also recorded reflections on how perceived authority, racial difference, and motherhood status could influence rapport, disclosure, probing, and other aspects of the interview process. When concerns arose, we documented adjustments made during data collection, such as rephrasing prompts, allowing more silence, or scheduling follow-up contact. We reviewed journal entries during team debriefings, refined interview materials as needed, and revisited interpretations to ensure they remained grounded in the dataset and consistent with cultural safety.

After data collection, we transcribed the interviews and invited women to review their transcripts for accuracy and completeness. We sent transcripts via secure email, and twelve women confirmed that the transcripts reflected their interviews, while ten did not respond. We used this process for transcript verification rather than for participant validation of thematic interpretations. During analysis, we used team expertise to evaluate and refine the developing themes and subthemes. We met weekly or bi-weekly via Zoom to examine interpretations, discuss discrepancies, and ensure the themes remained grounded in the data. We used field notes alongside interview transcripts to corroborate and contextualize the findings. To enhance transferability, we reported detailed demographic information and described the study setting, the roles of the researchers, and the analytic assumptions. We strengthened dependability by maintaining an audit trail that documented key methodological and analytic decisions.

## 3. Results

In this article, we examine how feeling unheard shaped women’s distress, trust, and care-seeking after perinatal loss and in a subsequent pregnancy. The narratives also reflected broader structural conditions that shaped clinical interactions and maternity care experiences. Three themes emerged: unheard and dismissed concerns, biased and stratified care, and perinatal loss follow-up gaps driving self-advocacy. Together, the themes and subthemes summarized in Table 3 indicate that dismissive communication, racialized and stratified care, and limited follow-up operated as interconnected processes that intensified distress, weakened trust, and shifted the burden of securing answers, continuity, and safety onto women themselves, particularly in subsequent pregnancies. Although these themes are not necessarily unique to Black women, this analysis shows how they were situated within U.S. maternity care systems that disproportionately shape Black women’s maternity care experiences.

### 3.1. Unheard and Dismissed Concerns

Women described feeling unheard in maternity care settings during and after perinatal loss and in subsequent pregnancies. Across interviews, women reported that healthcare providers minimized symptoms, delayed or did not respond to concerns, and engaged in interactions that felt emotionally detached. These experiences heightened vulnerability and distress, especially when women repeatedly raised concerns and their healthcare providers did not offer clear evaluation or follow-up care during or after perinatal loss. Women indicated that these patterns were present during the pregnancy that ended in loss and, for some, continued to influence how they approached maternity care in subsequent pregnancies. This theme is reflected in three subthemes: minimized symptoms and delayed action, nonresponsive communication in clinical encounters, and feeling unseen.

#### 3.1.1. Minimized Symptoms and Delayed Action

Women described seeking maternity care after noticing changes during pregnancy that raised concern. Several women reported contacting or presenting for maternity care more than once when symptoms continued or intensified. They recalled receiving reassurance from healthcare providers rather than additional evaluation or close follow-up care. Women often perceived these reassurances as minimizing the seriousness of what they were experiencing. They reported being told their symptoms were “normal,” even when they perceived that those symptoms were worsening or not resolving. Many women continued monitoring symptoms at home while uncertainty and worry persisted. One woman explained:

*“I told them that I was cramping. I told them that I was spotting. I told them that I had back pain, but they just kept telling me that’s normal.”*

Another added:

*“I had been noticing spotting. I was monitored but I ended up being sent home and they weren’t concerned about the spotting, so they just sent me home.”*

Pregnancy and postpartum concerns were sometimes addressed in ways that women experienced as minimizing, particularly when they reported bleeding during pregnancy or postpartum changes they did not perceive as normal. Some women recalled being reassured that symptoms were expected postpartum rather than receiving additional assessment or clear guidance. Women emphasized how difficult these interactions felt when their bodies signaled that something was wrong, and the reassurance did not align with their lived experience. One woman shared the following:

*“‘Oh, everything is okay. It’s just normal bleeding from being pregnant.’ I was like, ‘That’s not normal bleeding.’”*

#### 3.1.2. Nonresponsive Communication in Clinical Encounters

Women described interactions with healthcare providers that felt scripted and marked by limited engagement with their concerns. They reported that providers asked questions without attending to their responses. Others recalled receiving serious information without clear communication, preparation, or support. One woman stated:

*“She didn’t listen to anything that I said. It was like, ‘Well, how are you feeling? Uh-huh, yeah, okay, sure, yeah.’ Like, she was just asking me, and not even paying attention to my answer, just going through the motions.”*

Communication about serious findings often lacked clear explanation and guidance, contributing to women’s experiences of nonresponsive care. Several women recounted receiving devastating information quickly and with limited explanation of what it meant or what would happen next. Women emphasized leaving these conversations without clear guidance or written information to support understanding and decision-making. One woman described the following:

*“He looks at you and says, ‘There’s an obstruction here, after that, everything gets really complicated.’ We were stuck with no information, nothing printed out. He didn’t even write down the name of the condition.”*

#### 3.1.3. Feeling Unseen

Women described healthcare encounters that left them feeling unseen during pregnancy and after their loss. Rather than experiencing care that acknowledged the significance of the event, some women described interactions that felt impersonal, with little emotional recognition. Women reported that healthcare provider communication felt detached, and they perceived that the focus on routine tasks left little room for acknowledgment, clear information, and support. Some women described feeling overlooked in the maternity setting, either through limited proactive assessment or by being treated as though their continued presence in the hospital required justification. One woman shared:

*“There wasn’t even like a congratulations, oh my God I’m so happy for you. There was nothing… Again, the, this is a patient, I’m marking this patient off of my to-do list.”*

### 3.2. Biased and Stratified Care

Women described inequities in maternity care that they perceived were shaped by systemic racism and stratification within maternity settings. Across interviews, many women reported that racialized assumptions influenced how healthcare providers assessed symptoms, responded to pain, and communicated about risk, although these experiences were not universal across all accounts. Women linked these experiences to multiple aspects of care, including symptom evaluation, pain treatment, communication, hospital access, and care in subsequent pregnancies, indicating that these concerns were not limited to isolated encounters. Women described how insurance coverage affected access, continuity, and perceived quality of care, contributing to the sense that care differed by race and insurance status. These experiences were associated with heightened psychological distress, including anxiety, particularly in subsequent pregnancies after loss. This theme is reflected in three subthemes: racialized assumptions, insurance-stratified care coverage, and racism-related psychological distress.

#### 3.2.1. Racialized Assumptions

Many women reported situations in which they believed race shaped how healthcare providers interpreted their concerns and responded to them, although the specific forms these experiences took varied across accounts. These accounts extended across pregnancy, loss, and postpartum care and included concerns about symptom credibility, pain assessment, and provider suspicion. They perceived that healthcare providers minimized reported symptoms, treated pain as less credible, or approached them with suspicion instead of pursuing further evaluation. A smaller subset of women also described pain during procedures being disregarded. One woman recounted asking the anesthesiologist to stop an epidural because it was so painful, but she felt that her request was ignored, and the procedure continued. Experiences like these led some women to believe that their healthcare providers gave them less attention and took their concerns less seriously than those of White patients. They also linked these patterns to communication and clinical decision-making during pregnancy, loss, and postpartum care. One woman shared:

*“Doctors and nurses are more likely to pay attention to Caucasians. They just kind of tell us, like, ‘Oh, chalk it off;’ whereas a different race, they’re more like, ‘Oh, well, let me look into it.”*

Clinical conversations sometimes conveyed suspicion and implied blame, particularly through the way healthcare providers questioned women about stigmatized behaviors. Women interpreted these interactions as signs that healthcare providers doubted their credibility or responsibility. They often linked these experiences to questions about substance use or other health risk behaviors. Women perceived moral judgment and disproportionate scrutiny tied to race rather than standard screening or supportive assessment. One woman explained:

*“He’d ask me questions that they would only ask African American people I’m pretty sure. He asked, ‘Well, did you do drugs with this baby?’ ‘Well did you smoke any tobacco with this child?’”*

#### 3.2.2. Insurance-Stratified Care Coverage

Insurance coverage shaped how women interpreted the quality of maternity care they received. Women connected insurance status not only to provider effort, but also to hospital choice, continuity of care, and the boundaries of available services. Several women linked public insurance to feeling that healthcare providers invested less effort and were less responsive during pregnancy and around the time of loss, while others emphasized restricted hospital options or limits on continuity of care. They also reported that insurance coverage influenced where they could give birth, including being unable to deliver at a preferred hospital because it was out of network or not covered. In those situations, women described receiving care at facilities covered by their insurance and perceived differences in how they were treated. Women also noted that coverage could shape the boundaries of care, reinforcing the sense that public health insurance influenced both access and the overall care experience. One woman shared:

*“I felt like because I was on Medicaid you get crap doctors and they don’t really put forth the effort.”*

Another woman added:

*“Because of my insurance, it would not cover that hospital as far as delivery.”*

#### 3.2.3. Racism-Related Psychological Distress

Many women described psychological distress during the pregnancy that ended in loss and reported that these feelings often carried into a subsequent pregnancy, although not all women linked that distress to racism in the same way. Across accounts, women first described distress related to the emotional aftermath of perinatal loss, including fear, uncertainty, and unresolved worry after acute care experiences. They reported fear and uncertainty during acute care experiences, including being instructed to go home to miscarry, waiting for care while grieving, or experiencing complications without clear reassurance. Some women also reported anxiety in subsequent pregnancies and described efforts to manage these feelings while facing uncertainty. Many described these loss-related experiences as emotionally difficult and reported that they contributed to ongoing distress. Women also reported worry about what would happen next and whether they were safe. Some women described awareness of racial inequities as intensifying distress that was already present after perinatal loss, particularly in subsequent pregnancies. This awareness included Black maternal mortality and community-level knowledge of disproportionate risk. One woman explained:

*“They came back and said, ‘No heartbeat detected.’ And then they tell me, ‘Just go on home and when you start bleeding you’re gonna miscarry.’ Are you serious?! I was afraid to go to the bathroom.”*

Another woman described how awareness of Black maternal risk intensified fear during a subsequent pregnancy:

*“Am I gonna be healthy during this pregnancy? Am I gonna make it full-term?… Like, a lot of stressors especially with all the African American women dying in birth… Like am I gonna live?”*

### 3.3. Perinatal Loss Follow-Up Gaps Driving Self-Advocacy

Women described gaps in follow-up care during and after perinatal loss and in subsequent pregnancies that left them feeling responsible for coordinating care and securing information on their own. Across interviews, women described follow-up care from their healthcare providers as limited, delayed, and unclear, which contributed to uncertainty and heightened stress. In response, many women described advocating for themselves by repeatedly seeking care, requesting clarification, and pushing for evaluation when their healthcare providers did not address their concerns. Some described changing healthcare providers or relocating care to find more responsive, culturally safe support. This theme is reflected in three subthemes: limited perinatal loss follow-up, self-advocacy for care, and transitioning to culturally safe healthcare providers.

#### 3.3.1. Limited Perinatal Loss Follow-Up

Women described follow-up care during and after perinatal loss as limited, delayed, or unclear. They reported leaving clinical encounters without proactive outreach, clear guidance, or reassurance from healthcare providers after discharge. Some described this absence of follow-up care during and after perinatal loss as reinforcing feelings of being unheard and left to manage physical recovery, grief, and unanswered questions on their own. Women also linked these gaps to heightened uncertainty and distress during the period after loss and, for some, into subsequent pregnancies. One woman shared:

*“I didn’t have any therapy, I didn’t have any, like, follow-up care, even from the hospital. Like, I don’t know where my baby’s body is, what they did with him, uh, I was never officially given a reason for the loss. There was nothing. I was just released from the hospital and that was pretty much it.”*

#### 3.3.2. Self-Advocacy for Care

Limited follow-up care during and after perinatal loss, along with unresolved concerns, often required women to advocate for care on their own. They reported repeatedly asking questions, seeking clarification, and returning for evaluation when they felt something was wrong. Some women described self-advocacy as necessary to obtain information, reassurance, or additional assessment in subsequent pregnancies. Women also framed these efforts as a response to feeling unheard and to navigating maternity care environments in which they could not assume their concerns would be acted on. Several linked self-advocacy to protecting their own wellbeing and the wellbeing of a subsequent pregnancy. One woman shared:

*“During the pregnancy, do not be afraid to ask questions. Definitely go with a list of questions. Never leave without asking at least one question, and do not be afraid to speak up for yourself. If you want to know what is going on, tell them ‘tell me what’s going on.’ If you feel something isn’t right. Don’t be afraid to go to the emergency room and say; ‘Listen, something don’t feel right. Um, I haven’t felt my baby move in like 10 h. I need to check my baby.’”*

Women also described self-advocacy as pushing for immediate evaluation when they believed something was wrong with their pregnancy. They emphasized that asking questions was not enough; they had to insist on being seen when symptoms or fear persisted. Women framed this as a difficult but necessary response to prior experiences of perinatal loss and to maternity care interactions in which reassurance alone did not feel sufficient. One woman explained:

*“Ask every single question and double ask every single question. And if at any point you feel something is wrong, go straight to triage and demand that you’re looked at and that you are seen.”*

#### 3.3.3. Transitioning to Culturally Safe Healthcare Providers

Changing healthcare providers or maternity care settings became one way that women responded to feeling unheard during and after perinatal loss and during a subsequent pregnancy. They described seeking out different healthcare providers, hospitals, or maternity care systems when prior interactions left them feeling dismissed, unsupported, or emotionally unsafe. Some women reported that these decisions were shaped by a desire for greater continuity of care and for maternity care environments that felt more affirming after perinatal loss. In a smaller number of contrasting accounts, women described these new care settings as supportive and protective because they offered clearer follow-up, more responsive communication, and a greater sense of cultural safety than women had experienced previously. They linked these transitions to cultural safety, including feeling more comfortable with healthcare providers and staff who looked like them and whom they believed could better understand their experiences. One woman shared:

*“And my new OB/GYN is at a whole different hospital, a whole different outlook. I just kinda like did everything new. I think for me, like being an African American woman I want a doctor that looks like me. I want to be able to go to someone who maybe can relate a little bit more to me.”*

Another woman explained:

*“I have a new doctor now. Not only are they close to my house, but everybody that works in their office, they look just like all Black women, you know? Like, culturally, I feel comfortable here.”*

## 4. Discussion

Using semi-structured interviews, this study found that Black women who experienced perinatal loss often navigated maternity care in ways that left them feeling unheard, dismissed, and insufficiently supported during perinatal loss and in subsequent pregnancies. Racialized assumptions, insurance-stratified care, and limited follow-up care during and after perinatal loss shaped these experiences and intensified psychological distress while undermining trust and continuity of care. As a result, many women assumed responsibility for monitoring symptoms, securing information, and advocating for safe, responsive, and culturally affirming maternity care. Although systemic racism provided the central interpretive lens for this analysis, these findings should not be read as suggesting that racism operated in isolation. Women’s accounts also pointed to interacting conditions, including insurance-stratified access, fragmented follow-up systems, continuity gaps, and other structural barriers that shaped how concerns were heard, acted on, or deferred within maternity care settings [2,3,4,21,22,23,24,36].

### 4.1. Unheard Concerns in Pregnancy and Loss

Feeling unheard shaped women’s experiences of maternity care across the continuum of pregnancy, perinatal loss, and in a subsequent pregnancy. These findings suggest that dismissive and emotionally detached interactions reflect more than isolated breakdowns in care. They revealed a broader pattern of patient–provider dynamics that heightened uncertainty and weakened trust in maternity care when women raised serious concerns. Similar patterns have appeared in prior work with Black women [3,53]. Poor patient–provider interactions have undermined perinatal care experiences and shaped how women interpreted the care they received. Perceived discrimination in prenatal care has further contributed to those experiences [53]. Breakdowns in continuity of care have also weakened trust during severe obstetric complications, particularly when women needed clear guidance and consistent follow-up from their healthcare providers [54]. The absence of individualized care may deepen that harm by leaving women feeling reduced to “just a number” rather than recognized as a person with meaningful concerns [55]. In these circumstances, minimized symptoms and delayed action did more than postpone evaluation. They likely weakened trust and left women to monitor potential warning signs on their own at moments when timely assessment might have mattered most [56].

Nonresponsive communication intensified the distress surrounding perinatal loss. Women described providers who asked questions without engaging with what they reported and who delivered serious findings without the explanation or preparation needed to make sense of what was happening. Poor patient–provider interactions and perceived discrimination in prenatal care have similarly shaped Black women’s perinatal experiences, while gaps in continuity of care and support have further weakened trust during severe obstetric complications [53,54]. These gaps may become even more consequential during perinatal loss, when women leave maternity care settings without clear explanations about clinical findings or what will happen next. Under these circumstances, information and responsive support may shape whether care feels person-centered, particularly because person-centered maternity care emphasizes communication, autonomy, and supportive engagement [57]. When women leave these encounters without guidance about what happened or what will happen next, grief, anxiety, and fear may intensify in ways that extend beyond the perinatal loss itself [4]. These unresolved experiences may also carry into subsequent pregnancies, heightening vigilance, weakening trust, and increasing the need for reassurance [58].

Feeling unseen affected women’s experiences during pregnancy and after perinatal loss. Women wanted healthcare providers to recognize the significance of their pregnancy and loss. Instead, some women described care that focused on routines and tasks. This approach left little space for emotional recognition or a sense of being seen as a person. It also fails to reflect respectful maternity care and leaves little room for person-centered acknowledgment. Neglect and poor interpersonal treatment remain common in U.S. maternity care [59]. Evidence remains limited on how Black women experience a lack of acknowledgment during and after perinatal loss and in subsequent pregnancies. Black women also describe being listened to, receiving follow-up care during and after perinatal loss, and being treated with respect as core features of high-quality maternity care [60]. When maternity care leaves women feeling unseen during perinatal loss, it may shape how they enter care in a subsequent pregnancy. Some women responded by switching healthcare providers in search of care that felt more attentive and engaged.

### 4.2. Biased and Stratified Care Across Loss and Subsequent Pregnancy

Women’s accounts suggest that racialized assumptions may shape how healthcare providers interpret Black women’s concerns and determine whether further evaluation is needed. Judgment and stereotyping also mark Black women’s clinical encounters in maternity care [61,62,63]. Disrespect, exclusion from decisions about care, and suspicion further reflect these patterns [58,59,64]. These encounters also expose power imbalances within the patient–provider relationship, where healthcare providers often control information, determine whose symptoms are treated as credible, and shape how fully Black women are included in decisions about evaluation and treatment [6,61]. In women’s accounts, these power imbalances appeared in the form of minimized symptoms, discounted pain, and clinical interactions that women perceived as suspicious or dismissive. For Black pregnant women, that imbalance can be dangerous since maternity care often depends on prompt responses to patient-reported symptoms. These accounts suggest that when healthcare providers minimize concerns or filter symptoms through racialized assumptions, they may miss opportunities to reevaluate symptoms or escalate care [8,65]. Prior literature further suggests that biased pain assessment can affect how healthcare providers interpret and respond to Black women’s symptom reports, including pain, which often functions as an important clinical signal in maternity care [66]. Although this study did not measure delayed recognition of complications or adverse pregnancy outcomes directly, prior research links biased evaluation and delayed response to traumatic birth, severe maternal morbidity, and other adverse maternal and infant outcomes [2,8,58,66].

Women’s accounts suggest that insurance-stratified care may shape where Black women seek care and which healthcare providers they can access. It may also influence whether care feels responsive and safe. Women linked public insurance, such as Medicaid, with restricted hospital options and fewer choices during pregnancy and around the time of loss. They also believed their insurance coverage influenced how much effort healthcare providers put into their care. This pattern affected more than maternity care access. It also influenced whether women felt safe, were believed about their symptoms, and were valued within healthcare encounters. Structural barriers of this kind can delay and disrupt continuity of maternity care [2,36,56,67]. In turn, Black women may have fewer opportunities for timely and equitable maternity care across the perinatal period. Collaborative and culturally centered prenatal and postpartum care can improve patient safety and clinical outcomes [36]. Reducing structural barriers and strengthening continuity of maternity care are therefore critical steps in addressing stratified care and improving maternal and infant health outcomes after perinatal loss and in subsequent pregnancies. These patterns suggest that inequitable care experiences were shaped not only by racialized assumptions in clinical encounters, but also by the organizational and access conditions through which care was delivered, including restricted provider choice, discontinuity across settings, and limited follow-up infrastructure [36,56,67].

Psychological distress during and after perinatal loss may extend into a subsequent pregnancy, where fear about safety persists. Limited reassurance during maternity care may intensify distress during the perinatal loss experience and leave women carrying unanswered questions into a subsequent pregnancy. This distress may weigh especially heavily on Black women, who also face racial inequities in maternal health outcomes. Black maternal mortality rates sharpen this fear. Systemic racism contributes to the conditions that place Black women at greater risk of maternal death [2,68,69]. It also influences healthcare policy and access to maternity care during pregnancy, birth, and the postpartum period [68,69]. Throughout pregnancy and birth, systemic racism affects maternity care and the clinical management of pregnancy-related symptoms and complications [2,68,69]. Against this backdrop, awareness of Black maternal mortality may heighten fear about personal safety and fetal wellbeing after a prior perinatal loss. In this study, some women explicitly linked that fear to their awareness of racial inequities in maternal health. Prior literature helps situate those concerns within inequitable maternity care systems [2,8,68,69]. This fear reflects more than individual emotional distress and may also represent a reasonable response to lived experience, community knowledge, and maternal health systems shaped by racial inequity [2,8]. Racism-related distress in a subsequent pregnancy reflects not only the emotional aftermath of perinatal loss, but also the reality that Black women enter pregnancy within maternal health systems that expose them to unequal risk [2,8,36].

### 4.3. When Perinatal Follow-Up Gaps Required Self-Advocacy

Limited follow-up care during and after perinatal loss may prolong uncertainty and intensify psychological distress when women leave care without clear answers and without timely contact or guidance from healthcare providers [70,71]. When follow-up care is absent during and after perinatal loss, unanswered questions may extend beyond discharge and carry into a subsequent pregnancy [70,71]. For Black women, these gaps may carry additional harm because they can fall on top of higher maternal mortality and broader disadvantage in maternal health care [2,68,69]. Mental health concerns may be especially easy to miss under these conditions. Perinatal loss is associated with elevated depression and posttraumatic stress symptoms, yet many bereaved women do not receive treatment [2,72]. Black women also face persistent gaps in perinatal mental health care. Compared with White and Latina women, they experience higher rates of anxiety, depression, and posttraumatic stress [73,74,75,76]. These differences may be linked to barriers in accessing care and to the lack of care that adequately addresses the intersection of health and sociocultural experiences across minoritized groups [77]. Their mental health needs are also less likely to be identified and treated [73]. Follow-up care is also less likely to continue over time [73,78,79]. Systemic racism contributes to gaps in diagnosis, treatment, and access to quality care [80]. For women experiencing perinatal loss, these gaps may do more than delay answers. Women’s accounts suggest that limited follow-up may also leave distress unrecognized during a period of heightened vulnerability, while prior literature indicates that untreated distress and disrupted follow-up can extend into a subsequent pregnancy [72,78,80].

This pattern suggests that self-advocacy became necessary when women could not trust healthcare providers to listen or act on their concerns. Similar findings align with prior work in which Black women describe self-education and self-advocacy as responses to racism, mistreatment, poor communication, and unsupportive care [77,81,82]. In that sense, self-advocacy may serve as one of the few immediate safeguards available in maternity care [83,84,85], creating another opportunity for evaluation when healthcare providers dismiss symptoms. It can also help women resist pressured interventions and protect their right to clear information and informed consent before obstetric procedures [83,84,85]. At the same time, this pattern shows how healthcare systems shift the work of securing safe maternity care onto women themselves, which may also carry psychological costs. Prior work suggests that expectations of strength and self-management may make it harder for Black women to disclose distress or seek mental health support after perinatal loss [52]. Self-advocacy in maternity care may therefore coexist with pressure to cope privately, which may deepen isolation and make distress less visible to healthcare providers.

Previous research shows that Black women report more positive prenatal and postpartum care when they experience continuity of care, collaborative relationships, and culturally centered maternity care [36]. Other studies also show that continuity of care and provider racial concordance are associated with more person-centered pregnancy and birth experiences among Black women [61]. For many Black women, a preference for a racially concordant prenatal provider reflects prior experiences of racism, discrimination, and race-related stress [86]. It may also reflect a desire for respectful communication after perinatal loss. In this study, some women associated racially concordant care with greater comfort and a stronger expectation of being understood. However, racial concordance should not be interpreted as equivalent to cultural safety. Rather, women’s accounts and prior literature suggest that cultural safety depends more fundamentally on respectful communication, trust, clinical competence, and follow-through on women’s concerns [36,87]. Some women may also seek care from providers who they believe will better understand the effects of bias and discrimination. These preferences also point to what may be at stake when women do not experience that kind of maternity care. By contrast, racial discordance may contribute to poorer communication [87]. It may also disrupt the exchange of information and limit shared decision-making. These dynamics can reduce the support and respect women experience in maternity care. Women are more likely to experience care as safe when providers offer respect, build trust, and demonstrate clinical competence [36,87,88]. In this study, changing healthcare providers reflected the work women undertook to find safety and continuity in maternity care systems that did not reliably provide affirming care.

### 4.4. Strengths and Limitations

This study offers robust qualitative evidence with several notable strengths. It provides in-depth qualitative insight into Black women’s experiences of systemic racism in maternity care settings, including healthcare provider bias, during and after perinatal loss, and in subsequent pregnancies. We used semi-structured interviews to elicit rich narratives about how women felt unheard, navigated biased and stratified care, and addressed follow-up care gaps by advocating for themselves. We recruited women from multiple U.S. regions and with varied socioeconomic and insurance coverage, which broadened the perspectives represented. We applied a rigorous, stepwise analytic approach and used field notes to add descriptive detail. We also conducted regular analytical debriefings to refine codes and themes, thereby strengthening credibility and trustworthiness. Together, these strengths contribute evidence in an area where research remains limited. They also underscore the need for culturally safe, equity-oriented maternity care that confronts systemic racism and improves communication, follow-up care, and support during and after perinatal loss.

This study has several limitations. The geographic distribution of the sample was concentrated in the Midwest and Eastern parts of the U.S., which may limit transferability to Black women in other regions, including the South, where political environments and availability of maternity resources can differ. Thirteen interviews took place by telephone, which reduced our ability to capture nonverbal communication and may have limited contextual richness. The English-language requirement likely excluded women who do not read or speak English. In addition, as is typical in qualitative inquiry, the findings rely on self-reported narratives and reflect what women chose to share about sensitive maternity care experiences. Because this analysis drew on a larger qualitative dataset that required women to report stress-related circumstances, the sample may have been weighted toward women with greater distress or more adverse experiences. This sampling characteristic may limit transferability to women who experienced perinatal loss and subsequent pregnancy but did not describe stress-related circumstances in similar ways. We also did not capture interview timing in sufficient detail to report how long after the qualifying loss or subsequent pregnancy or birth each interview occurred. As a result, we could not examine how temporal distance may have shaped recall, interpretation, or meaning-making. We did not collect information on women’s sexual orientation or partner gender identity, which limits transferability to LGBTQ+ individuals and varied relationship configurations. The modest sample size and the scope of the current analysis did not allow for in-depth comparison across important sources of variability within the sample, including socioeconomic circumstances, insurance coverage, perinatal loss type, infertility history, or healthcare setting. As a result, we could not determine whether women’s experiences differed systematically across these dimensions, which may limit transferability to Black women receiving care in other clinical, social, or organizational contexts. For these reasons, we do not claim that the themes identified here are unique to Black women. Rather, we situate them within the experiences of this cohort and within maternity care environments that disproportionately shape Black women’s care in the U.S.

Although this analysis centered on systemic racism as the primary interpretive lens, we do not suggest that racism was the only factor shaping these maternity care experiences. Women’s accounts also reflected interacting structural and organizational conditions, including insurance-related access barriers, continuity gaps, and fragmented follow-up care. At the same time, we did not directly measure clinician workload, staffing constraints, institutional resources, or health literacy, so we cannot determine how these factors may have interacted with systemic racism in specific encounters. Future research should intentionally collect sexual orientation, partner gender identity, infertility history, and healthcare setting characteristics and recruit larger, more diverse samples. It should also examine variation in maternity care experiences by socioeconomic circumstances, insurance coverage, perinatal loss type, and pregnancy circumstances, as well as how systemic racism interacts with organizational, access-related, and other contextual factors across racial and ethnic groups. Future research should also document interview timing in relation to the qualifying loss and any subsequent pregnancy or birth so that analyses can better assess how timing may shape recall and meaning-making.

### 4.5. Implications and Recommendations

This study used semi-structured interviews to explore how Black women who experienced perinatal loss perceived and navigated patient–provider dynamics and systemic racism before and after loss and in a subsequent pregnancy. For these women, perinatal loss was not an isolated clinical event. They described it within maternity care systems marked by dismissive communication, racialized assumptions, insurance-stratified care, and limited follow-up after perinatal loss, all of which intensified distress and weakened trust. These conditions also extended into subsequent pregnancies, where women often carried greater fear, uncertainty, and responsibility for securing safe and responsive maternity care. Beyond individual clinical encounters, these findings have implications for maternity services and for perinatal public health efforts focused on continuity, follow-up, and linkage to supportive resources. Strengthening partnerships between maternity care, mental health care, and public health programs may improve follow-up care during and after perinatal loss and reduce fragmentation across the reproductive continuum. Taken together, these findings underscore the need for culturally safe maternity care that responds to Black women’s concerns, communicates clearly after perinatal loss, and supports both immediate and longer-term needs within racially inequitable systems. These findings also point to a translational agenda in which patient-reported experiences can inform structured postpartum and interpregnancy care models that reduce reliance on self-advocacy and improve continuity across maternity, bereavement, mental health, and community-based services [89,90,91,92]. Three priority areas for action align with the study themes: timely and responsive clinical communication when women raise concerns, structural and organizational changes that reduce biased and stratified care, and proactive follow-up and continuity of maternity care after perinatal loss and in subsequent pregnancies.

To address women’s experiences of unheard and dismissed concerns, maternity care should prioritize person-centered, trauma-informed, and culturally safe communication after perinatal loss and in subsequent pregnancies [36,80,87]. At the clinician level, healthcare providers should respond to concerns with timely evaluation rather than reflexive reassurance [8,72]. Communication during maternity encounters should be clear, compassionate, and specific [72,93]. When women receive care related to perinatal loss, healthcare providers should clearly explain what is known, what remains uncertain, and what the next steps in maternity care will be [71,72]. Women should also receive direct guidance about pregnancy or postpartum warning signs and when to return for urgent evaluation [72,94]. Respectful maternity care is especially important in this setting. Maternity care environments that validate Black women’s concerns and act on them may help rebuild trust and contribute to efforts to improve maternal and newborn outcomes. Black women have identified individualized communication, shared decision-making, and time with healthcare providers as central to safer and more acceptable maternity care [61,87]. These practices can be built into routine care through structured symptom assessment, explicit return precautions, and documented plans for follow-up care after discharge [72,94].

From a clinical obstetric perspective, these findings also point to the importance of structured care pathways after perinatal loss. Although management varies by gestational age, cause of loss, and care setting, evidence-based care after perinatal loss generally includes clear explanation of the diagnosis and available findings, attention to physical recovery, and guidance on next steps in care. Physical recovery may include assessment of bleeding, pain, blood pressure concerns, and signs of infection, along with counseling on lactation, contraception, and interpregnancy planning when appropriate. Follow-up care should also include review of assessment findings, attention to emotional wellbeing, and planning for a subsequent pregnancy [71,72,93,94].

To address perinatal loss follow-up gaps driving self-advocacy, health systems should prioritize structured follow-up during and after perinatal loss rather than relying on women to re-enter care on their own. These findings support standardized perinatal loss follow-up protocols that specify how and when services will re-establish contact after discharge [71,72,93]. One implication is that services may benefit from designating who will contact the woman after discharge and when that contact will occur [71,72]. A structured follow-up protocol may also specify the core elements of that contact, including symptom assessment, review of available and pending findings, emotional wellbeing screening, return precautions, and clear planning for subsequent care.

The follow-up encounter can review available findings and explain pending test results. It can also assess emotional wellbeing and provide clear next-step guidance [71,72]. Connecting women directly to bereavement and mental health support is also important, as referral alone may not be enough to help women access the care they need after perinatal loss [60,80]. Navigation support may be especially important for Black women who are already managing fragmented systems and unmet social needs [60]. Patient advocates, navigators, or designated hospital bereavement leads may help reduce the work women described after loss and in subsequent pregnancies [60,95]. Community-based doulas may provide another layer of advocacy and support, particularly when women anticipate mistreatment or do not want to navigate care on their own [95,96]. Medicaid reimbursement for doula services at the state level offers a promising policy model that may support efforts to improve birth outcomes and reduce health disparities [97]. Medicaid coverage of doula care can also support additional visits tailored to person-centered needs, including emotional support, grief support, self-advocacy, appointment attendance, and coordination with community resources [98]. Expanding this model as a nationwide Medicaid benefit may help ensure that Medicaid recipients across the U.S. have access to doula support during pregnancy, after perinatal loss, and in subsequent pregnancies. Women’s accounts of limited care during perinatal loss and delayed follow-up care afterward point to gaps in both clinical care and broader system support. Perinatal public health programs may support these needs by strengthening follow-up after perinatal loss and linking women to services in subsequent pregnancies. They can also link hospital care to community-based mental health, bereavement, and peer support resources that respond to community-identified needs [80,99].

Continuity and respectful maternity care are especially important in subsequent pregnancies because they address both the follow-up gaps and the mistrust women described after perinatal loss. Women in this study entered these pregnancies with fear, uncertainty, and uneven trust in the care they were receiving. Under these conditions, health systems can treat continuity of care as a safety strategy rather than a convenience [90,100]. Assigning a consistent healthcare provider or small care team in a subsequent pregnancy may improve communication and reduce the need for women to repeat their histories at every encounter [36,100]. It may also improve recognition of prior perinatal loss, ongoing distress, and new symptoms that require evaluation [90,100]. Women also described changing healthcare providers in search of care that felt safer and more affirming. These findings further suggest that placing full responsibility on patients to find culturally safe care may deepen the burdens women described. Referral pathways should make it easier to transfer care after a harmful encounter and easier to maintain continuity of care in a subsequent pregnancy [36,61]. Options for racially concordant care may matter when available, but concordance alone is not enough [86,87]. Women are more likely to feel safe when they receive respectful, trusting maternity care. Care is more likely to feel affirming when competence and cultural safety are also present [36,87,88].

To address biased and stratified care, maternity systems should prioritize training that strengthens bias recognition, cultural safety, and respectful maternity care [87,101]. Maternity settings can treat racial bias as a patient safety problem and address it through routine quality improvement work. Health systems can provide ongoing education in cultural safety and bias recognition [5,87]. This education can also address respectful maternity care, trauma-informed care, and informed consent [5,87]. It can also help healthcare providers communicate more effectively during urgent or emotionally charged encounters [5,101]. Training can include case review of delayed or dismissive encounters [72]. Education can also include structured reflection on racialized clinical judgment [5,72,87,101]. Role-play can prepare healthcare providers to recognize pregnancy warning signs early and respond promptly [72,101]. Medical schools, residency programs, and other health professions programs can begin this work early by integrating these topics into core curricula rather than leaving them to one-time workplace sessions. Adverse event review can also help hospitals identify when healthcare providers dismiss patient concerns [5,101]. Such reviews can also show when healthcare provider responses are delayed and when follow-up care during and after perinatal loss breaks down [5,101].

Policy action is also needed to address the structural conditions that contributed to biased and stratified care and to fragmented follow-up after perinatal loss. Policy can improve coordination and reduce fragmentation, so women do not have to arrange care on their own [80]. It can also hold maternity care systems accountable for responsive, respectful, and culturally safe care after perinatal loss. Policy can also establish consistent standards during pregnancy, after perinatal loss, and in subsequent pregnancies [93,100]. Such standards could include timely evaluation of symptoms and concerns after perinatal loss, as well as clear follow-through on test results, referrals, and next steps in care. Quality improvement efforts can track follow-up care during and after loss, timeliness of evaluation, and access to bereavement and mental health services [72,80,101].

The findings also support a need for stronger bereavement and mental health care after perinatal loss. For many women, the effects of loss extended beyond discharge and into a subsequent pregnancy. Health systems can respond by offering accessible bereavement and mental health support without unnecessary barriers to care [60,80]. They can also provide proactive scheduling, repeated offers of follow-up care, and peer or community-based support [80,95,99]. These services may help women who leave maternity care without clear answers and later enter a subsequent pregnancy with fear about their own safety. Together, these recommendations prioritize responses to the three central patterns identified in the study: women’s concerns being dismissed, care being experienced as biased or stratified, and women being left to navigate follow-up largely on their own.

Building on these findings, future research should move beyond descriptive accounts and evaluate structured, multidisciplinary interventions in real maternity care settings [100]. Priority areas include standardized follow-up and bereavement care pathways that reduce the burden on women to re-enter maternity care on their own [101]; integrated mental health, navigation, and doula-based support models that may improve emotional recovery and care coordination after perinatal loss [89,100]; continuity-of-care models in subsequent pregnancies that may strengthen trust, communication, and timely evaluation of new concerns [100]; and training and quality-improvement strategies that address bias recognition, cultural safety, and respectful maternity care at the clinician and systems levels [100]. Future intervention studies should assess not only psychological outcomes, such as grief, anxiety, depression, posttraumatic stress, and perceived support, but also care-process outcomes, including follow-up completion, understanding of evaluation findings, healthcare engagement, and continuity across care settings, as well as maternal health outcomes in subsequent pregnancies [89,90,100]. Longitudinal studies can clarify the effects of gaps in follow-up care during and after perinatal loss on trust and healthcare engagement. Researchers can also examine how those gaps affect care needs in subsequent pregnancies [72,91]. Continuity of care models for subsequent pregnancies also warrant evaluation [72,91]. Culturally responsive bereavement and mental health support models deserve further study. This includes peer-led initiatives and integrated mental health care [52]. Doula support and other community-based advocacy models may also ease care navigation after perinatal loss [95,96]. These models may also strengthen communication and support continuity of care in subsequent pregnancies [95,96]. Training approaches that strengthen cultural safety also merit evaluation [52,92]. Researchers can also examine approaches that improve bias recognition. They can also evaluate training that supports respectful, trauma-informed maternity care [52]. This work can clarify whether such training improves patient trust and healthcare engagement [52,92]. It can also assess whether these approaches are associated with more equitable maternal health outcomes [92]. Researchers can also examine how these strategies function across different perinatal loss types and pregnancy circumstances. They can also assess how care needs shift in subsequent pregnancies [91,94]. Studies can evaluate how surveillance systems and prevention efforts address community-identified needs during and after perinatal loss. Researchers can also examine how policy reforms respond to those needs. By examining scalability and sustainability, future work can identify effective models and guide efforts to reduce inequities in care after perinatal loss. Future studies should also include women from different racial and ethnic groups, while still attending to the racialized clinical and structural conditions that shaped the experiences described in this study.

## 5. Conclusions

This study highlights how systemic racism and patient–provider dynamics shaped Black women’s maternity care experiences during and after perinatal loss and into a subsequent pregnancy. Women identified feeling unheard, biased and stratified care, and perinatal loss follow-up gaps as interconnected experiences. Together, these experiences intensified distress and shifted the work of securing answers, safety, and continuity of care onto women themselves. These findings also show that self-advocacy often emerged as a response to maternity care systems that did not reliably listen, explain, or follow through after perinatal loss. In turn, these gaps shaped how women entered a subsequent pregnancy, often with greater fear, reduced trust, and a heightened need to secure safe and responsive care on their own. This analysis is relevant to both maternity services and public health, suggesting that efforts to improve care after perinatal loss must address more than access to care alone. These intersecting clinical and structural harms affect both psychological and physical wellbeing. These findings suggest that timely responses to women’s concerns and clear communication remain central to maternity care after perinatal loss. They also point to the importance of compassionate follow-up care during and after perinatal loss and culturally safe support across the reproductive continuum. The findings further indicate that stronger bereavement and mental health support, better continuity of care in subsequent pregnancies, and training that strengthens bias recognition and cultural safety may be important components of efforts to reduce disparities. These findings draw on the experiences of Black women and should be understood within the racialized clinical and structural conditions that shaped those experiences, rather than as intrinsic to them. They point to actionable changes that women in this study identified as meaningful. Women emphasized responsive care, clear guidance after perinatal loss, and consistent follow-up care from healthcare providers. They also desired clinical relationships grounded in trust, respect, and continuity of care. Further research is needed to examine how these strategies may work across racial and ethnic groups and how women’s maternity care needs may differ by perinatal loss type, pregnancy circumstances, and experiences in a subsequent pregnancy.

## Figures and Tables

**Table 1:** Women’s Demographics and Broader Reproductive and Loss History (*n* = 22).

Domain	Characteristic	Value
**Demographic characteristics**	Age, mean (range), years	34.2 (18–48)
	Region	Midwest (10); South (6); Northeast (3); West (2); Central (1)
	Housing	Renting (13); Homeowner (9)
	Education	<High school (1); High school (4); Some college (8); College degree (9)
	Annual income	<$20,000 (7); $20,000–$30,000 (5); $30,001–$40,000 (1); $40,001–$50,000 (2); $60,001–$70,000 (1); >$80,000 (6)
	Relationship status	Married (9); Single (7); Divorced (4); Engaged (1); In a relationship (1)
	Employment status	Employed (16); Unemployed (5); Full-time student (1)
**Broader self-reported reproductive and loss history**	Healthcare coverage	Federal–state program (12); Private (10)
	Number of pregnancies, median (range)	4 (1 to 10 or more)
	Number of perinatal losses, median (range)	2 (1 to 4 or more)
	Gestational age at any self-reported loss	<14 weeks (14); ≥14 weeks (19)
	Newborn age at any self-reported loss	≤28 days (3)
	Infant age at any self-reported loss	>28 days (2)

Note. Women’s demographic information is drawn from a larger qualitative dataset [3] and is presented here in a condensed, reformatted form for this article. Frequencies are presented as n. Categories related to perinatal loss characteristics may exceed the total sample size because some women experienced more than one loss. Loss-history variables reflect all self-reported prior perinatal and infant loss experiences and are not limited to the qualifying loss used for study eligibility.

**Table 2:** Key Topics and Prompting Questions.

Topic	Purpose of Inquiry	Prompting Questions
**Reasons for Participation**	To understand what led women to take part in the study and what they hoped to contribute by sharing their experiences.	What led you to participate in this study?
**Everyday Stress and Sources of Support**	To explore daily stressors, how those stressors shaped pregnancy experiences, and where women sought emotional support.	What kinds of stress or pressure are part of your day-to-day life? How have these stressors affected this pregnancy or a previous pregnancy? Who do you talk with when you need support for stress or emotional concerns?
**Perinatal Loss Experience**	To examine women’s recollections of the pregnancy loss, the care they received, factors that shaped the experience, and the support available afterward.	Please tell me about the pregnancy that ended in loss. How would you describe the prenatal care you received during that pregnancy? What type of care or follow-up did you receive after the loss, and how well did it meet your needs? What circumstances most shaped your experience of the loss? What additional stressors made grieving more difficult after the loss? What helped you cope or find comfort after your baby died?
**Pregnancy After Loss**	To explore women’s experiences in a subsequent or current pregnancy, including emotions, challenges, prenatal care, and connection with the baby.	Please tell me about your current or most recent pregnancy. How did you feel when you learned you were pregnant again? What aspects of this pregnancy have been most difficult? How would you describe your connection with your baby during this pregnancy? What stressors have affected you during this pregnancy? How would you describe the prenatal care you received during this pregnancy? What emotions have stood out most during this pregnancy?
**Reflection and Messages for Others**	To invite women to reflect on their experiences and share insights they would want other mothers to hear after perinatal loss.	What would you want other mothers who have experienced loss to know from your experience?

Note. Prompts were drawn from a larger qualitative dataset [3] and are presented in condensed form to illustrate the main areas explored during the interviews.

**Table 3:** Themes, Subthemes, Definitions, and Illustrative Quotes (*n* = 22).

Theme	Subtheme	Definition	Illustrative Quote
**1. Unheard and Dismissed Concerns**	Minimized symptoms and delayed action	Women described symptoms being normalized, dismissed, or not acted on despite repeated concern during pregnancy or postpartum.	*“Like any symptom a pregnant woman is feeling it should be… People should care about it. I mean, it shouldn’t just be looked over as, ‘Oh, you’re fine, just go home and then, you know, you’ll be okay.’ Or… ‘that’s just what happens when you’re pregnant.’”*
	Nonresponsive communication in clinical encounters	Women described communication that felt dismissive, emotionally detached, or lacking in seriousness during clinical care.	*“I was lying down for about 30 min while he was telling me this devastating news with no positive outcome… we were stuck with no information, nothing printed out… he didn’t even write down the name of the condition.”*
	Feeling unseen	Women described care that felt impersonal and unrecognizing of the emotional significance of their loss.	*“When I got in there no one had told the nurse what happened. When she came in, she put the fetal monitor on me to hear the baby’s heartbeat… I can’t even explain to you what that made me feel like.”*
**2. Biased and Stratified Care**	Racialized assumptions	Women perceived that race shaped how healthcare providers interpreted pain, symptoms, and credibility.	*“It’s like when you are a pregnant Black woman… when you’re saying that you’re hurting, they’re thinking that you want medication more than that you actually are in pain. They’re thinking that you want to abuse, versus, like, you’re actually going through something.”*
	Insurance-stratified care coverage	Women described insurance status as shaping access, healthcare provider options, and the quality or continuity of care.	*“I need my insurance because my doctors don’t take Medicaid, and this is a high-risk doctor.”*
	Racism-related psychological distress	Women described fear, anxiety, and uncertainty in subsequent pregnancy, including distress intensified by awareness of racial inequities in maternal outcomes.	*“So, like I try not to think about the negative stuff sometimes, but it’s hard. Especially when you read about it, when you see about it. Are we gonna hear a heartbeat? … I’m constantly checking.”*
**3. Perinatal Loss Follow-Up Gaps Driving Self-Advocacy**	Limited perinatal loss follow-up	Women described follow-up after loss as limited, delayed, or unclear, leaving them to manage recovery and unanswered questions on their own.	*“As soon as I noticed I was miscarrying I called and I was able to talk to the triage nurse. I called multiple times, because I had a lot of questions about aftercare. I was always the one that initiated the contact.”*
	Self-advocacy for care	Women described needing to ask for more care, monitoring, or reassurance when prior loss history was not acted on.	*“We are following up, we have um, appointments with two different OBGYNs just to kind of have a new set of eyes look at our history, and just kind of talk us through some things.”*
	Transitioning to culturally safe healthcare providers	Women described changing providers or care settings to find more responsive, reassuring, or culturally comfortable care.	*“I went to a different high risk this pregnancy… my new high-risk doctor said he’s going to put a cerclage in anyways… and if I ever felt uncomfortable… they would have me come in as soon as possible.”*

Note. Themes and subthemes are drawn from the current analytic structure of the manuscript. Definitions are presented in condensed form from the Section 3. Illustrative quotes were selected from participant transcripts to represent the core meaning of each subtheme.

## Data Availability

The data from this study is not publicly available, as participants did not provide consent for sharing beyond the research team.
